# Expression of cocoa genes in *Saccharomyces cerevisiae* improves cocoa butter production

**DOI:** 10.1186/s12934-018-0866-2

**Published:** 2018-01-25

**Authors:** Yongjun Wei, David Bergenholm, Michael Gossing, Verena Siewers, Jens Nielsen

**Affiliations:** 1 Department of Biology and Biological Engineering, Chalmers University of Technology, 41296 Gothenburg, Sweden; 2 Novo Nordisk Foundation Center for Biosustainability, Chalmers University of Technology, 41296 Gothenburg, Sweden; 3 Novo Nordisk Foundation Center for Biosustainability, Technical University of Denmark, 2800 Kgs. Lyngby, Denmark; 4 Present Address: CAS-Key Laboratory of Synthetic Biology, Institute of Plant Physiology and Ecology, Shanghai Institutes for Biological Sciences, Chinese Academy of Sciences, Shanghai, 200032 China

**Keywords:** Yeast cell factories, Cocoa butter-like lipid, *Theobroma cacao*, TAG biosynthetic genes, Metabolic engineering, Synthetic biology

## Abstract

**Background:**

Cocoa butter (CB) extracted from cocoa beans (*Theobroma cacao*) is the main raw material for chocolate production, but CB supply is insufficient due to the increased chocolate demand and limited CB production. CB is mainly composed of three different kinds of triacylglycerols (TAGs), 1,3-dipalmitoyl-2-oleoyl-glycerol (POP, C16:0-C18:1-C16:0), 1-palmitoyl-3-stearoyl-2-oleoyl-glycerol (POS, C16:0-C18:1-C18:0) and 1,3-distearoyl-2-oleoyl-glycerol (SOS, C18:0-C18:1-C18:0). In general, *Saccharomyces cerevisiae* produces TAGs as storage lipids, which consist of C16 and C18 fatty acids. However, cocoa butter-like lipids (CBL, which are composed of POP, POS and SOS) are not among the major TAG forms in yeast. TAG biosynthesis is mainly catalyzed by three enzymes: glycerol-3-phosphate acyltransferase (GPAT), lysophospholipid acyltransferase (LPAT) and diacylglycerol acyltransferase (DGAT), and it is essential to modulate the yeast TAG biosynthetic pathway for higher CBL production.

**Results:**

We cloned seven GPAT genes and three LPAT genes from cocoa cDNA, in order to screen for CBL biosynthetic gene candidates. By expressing these cloned cocoa genes and two synthesized cocoa DGAT genes in *S. cerevisiae*, we successfully increased total fatty acid production, TAG production and CBL production in some of the strains. In the best producer, the potential CBL content was eightfold higher than the control strain, suggesting the cocoa genes expressed in this strain were functional and might be responsible for CBL biosynthesis. Moreover, the potential CBL content increased 134-fold over the control Y29-TcD1 (IMX581 *sct1*Δ *ale1*Δ *lro1*Δ *dga1*Δ with *TcDGAT1* expression) in strain Y29-441 (IMX581 *sct1*Δ *ale1*Δ *lro1*Δ *dga1*Δ with *TcGPAT4*, *TcLPAT4* and *TcDGAT1* expression) further suggesting cocoa GPAT and LPAT genes functioned in yeast.

**Conclusions:**

We demonstrated that cocoa TAG biosynthetic genes functioned in *S. cerevisiae* and identified cocoa genes that may be involved in CBL production. Moreover, we found that expression of some cocoa CBL biosynthetic genes improved potential CBL production in *S. cerevisiae*, showing that metabolic engineering of yeast for cocoa butter production can be realized by manipulating the key enzymes GPAT, LPAT and DGAT in the TAG biosynthetic pathway.

**Electronic supplementary material:**

The online version of this article (10.1186/s12934-018-0866-2) contains supplementary material, which is available to authorized users.

## Background

*Theobroma cacao*, also known as cacao tree or cocoa tree, is an evergreen tree distributed in tropical areas [1, 2]. Its seeds, cocoa beans, can be used for extraction of cocoa butter (CB), which is a raw material for chocolate production. With chocolate demand increasing, more CB is needed [2]. Considering that cocoa trees grow only in the tropics and that replacing tropical forest with cocoa trees is not acceptable, planting more cocoa trees is not the choice for increasing CB production [3]. Moreover, CB production is easily affected by climate change, pest harm and microbial disease [4, 5]. Therefore, CB supply is limited and insufficient, and developing other stable and sustainable sources of CB supply is of interest [1].

Triacylglycerols (TAGs) of 1,3-dipalmitoyl-2-oleoyl-glycerol (POP, C16:0-C18:1-C16:0), 1-palmitoyl-3-stearoyl-2-oleoyl-glycerol (POS, C16:0-C18:1-C18:0) and 1,3-distearoyl-2-oleoyl-glycerol (SOS, C18:0-C18:1-C18:0) composed of C16 and C18 fatty acids are the three main components in CB [6]. Plant-derived CB-like lipids (CBL, mainly composed of POP, POS and SOS), such as illipe butter, shea butter and kokum butter, can be used as CB equivalents, but they are extracted from tropical plants as well and their lipid production is also limited [6]. In general, yeasts produce TAGs which consist of C16 and C18 fatty acids as storage lipids, making them good candidates for CBL production [7]. However, in the model yeast *Saccharomyces cerevisiae*, the naturally occurring CBL content is quite low [8]. Even though several oleaginous yeasts contain high amounts of lipids and their lipids have been considered as potential CB substitutes before, the small amount of naturally occurring CBL hindered their application in chocolate production [8, 9].

**Fig. 1:**
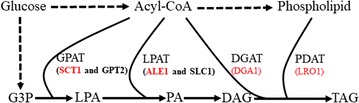
Three Enzymes, GPAT, LPAT and DGAT, determine the TAG structure in the TAG biosynthetic pathway. G3P glycerol-3-phosphate, LPA lysophosphatidic acid, PA phosphatidic acid, DAG diacylglycerol, TAG triacylglycerol, GPAT glycerol-3-phosphate acyltransferase, LPAT lysophosphatidic acid acyltransferase, DGAT acyl-CoA:diacylglycerol acyltransferase, PDAT phospholipid:diacylglycerol acyltransferase. *SCT1* and *GPT2* are GPAT genes, *SLC1* and *ALE1* are LPAT genes, *DGA1* is the DGAT gene, *LRO1* is the PDAT gene. The genes in red were deleted in some of the yeast strains used in this study

TAG biosynthesis is mainly catalyzed by glycerol-3-phosphate acyltransferase (GPAT), lysophospholipid acyltransferase (LPAT) and diacylglycerol acyltransferase (DGAT), which add acyl chains from acyl-coenzyme A (acyl-CoA) to the *sn*-1, *sn*-2 and *sn*-3 position of the backbone glycerol, respectively [10]. Therefore, besides sufficient C16 and C18 supply, modulation of yeast GPAT, LPAT and DGAT activity is essential for higher CBL production. There are two GPATs (Gpt2p and Sct1p), two LPATs (Slc1p and Ale1p), one DGAT (Dga1p) and one phospholipid:diacylglycerol acyltransferase, PDAT (Lro1p) that participate in TAG synthesis in *S. cerevisiae* (Fig. 1) [11–14]. The simultaneous deletion of either the two GPAT genes or the two LPAT genes (SLC1 and ALE1) is lethal to *S. cerevisiae*, but double deletion of *DGA1* and *LRO1* genes does not affect yeast growth [11, 15]. Single deletion of one GPAT gene or one LPAT gene alters yeast fatty acid profiles. For example, Sct1p has a preference towards C16 fatty acids and *sct1*∆ cells showed a different distribution of fatty acids in the phospholipids with less C16 fatty acids [11, 15]. Thus, disruption of some genes of the yeast TAG biosynthetic pathway and introducing corresponding heterologous genes would likely alter yeast CBL production. Overexpression of several synthesized cocoa TAG biosynthetic genes increased the CBL content in *S*. *cerevisiae*, but CBL production of these *S. cerevisiae* strains was still low [9]. As there are thirteen GPAT and nine LPAT genes in the cocoa genome [16, 17], deep and global characterization of cocoa GPAT and LPAT genes might reveal optimal cocoa genes responsible for CB biosynthesis, and expressing them in *S. cerevisiae* could improve CBL production [9].

Here, we cloned and expressed ten cocoa CB biosynthetic genes individually in *S. cerevisiae*, and compared the lipid production of the engineered yeasts. We also expressed some of the cloned cocoa genes together with two previously characterized cocoa DGAT genes in order to increase yeast CBL production. Moreover, we improved CBL production by metabolic engineering of the TAG biosynthetic pathway of *S. cerevisiae*.

## Methods

### Strains, plasmids, and media

The cloning host in this study was *Escherichia coli* strain DH5α. The *Lipomyces starkeyi* strain DSM 70296 was purchased from the culture collection of the DSMZ (Braunschweig, Germany). The *S. cerevisiae* strain CEN.PK 113-11C (*MAT*a *MAL2*-*8c SUC2 ura3*-*52 his3*-*Δ1*), which was kindly provided by P. Kötter [18] and *S. cerevisiae* strain IMX581 (*MAT*a *ura3*-*52 can1∆*::*cas9*-*natNT2 TRP1 LEU2 HIS3*) [19] were used in this study. *S. cerevisiae* CEN.PK 113-11C was used to express cocoa genes and homologous recombination was used to construct *S. cerevisiae* CEN.PK 113-11C-derived strains (Additional file 1: Figure S1). The plasmids pMEL10 and pMEL13 [19] were used for construction of strains derived from IMX581. All primers used to construct the yeast strains are listed in Additional file 1: Table S1, and all yeast strains constructed and used in this study are listed in Table 1. The strains YJ0 and SYJ0 were the same, but they were constructed and tested at different time as the control strain (Table 1).

**Table 1:** List of strains used in this study

Strains	Parent strains	Expression plasmids	Properties
YJ0	CEN.PK 113-11C [18]	pBS01A	Empty plasmid pBS01A
YJ-G03	CEN.PK 113-11C	PYJ-G03	*TcGPAT3* expression
YJ-G04	CEN.PK 113-11C	PYJ-G04	*TcGPAT4* expression
YJ-G05	CEN.PK 113-11C	PYJ-G05	*TcGPAT5* expression
YJ-G09	CEN.PK 113-11C	PYJ-G09	*TcGPAT9* expression
YJ-G10	CEN.PK 113-11C	PYJ-G10	*TcGPAT10* expression
YJ-G12-3	CEN.PK 113-11C	PYJ-G12	*TcGPAT12* expression
YJ-G12-4	CEN.PK 113-11C	PYJ-G12-4	*TcGPAT12*-*4* expression
YJ-L03	CEN.PK 113-11C	PYJ-L03	*TcLPAT3* expression
YJ-L04	CEN.PK 113-11C	PYJ-L04	*TcLPAT4* expression
YJ-L05	CEN.PK 113-11C	PYJ-L05	*TcLPAT5* expression
SYJ0	CEN.PK 113-11C	pBS01A	Empty plasmid pBS01A, same as YJ0, but SYJ0 was constructed and tested at a different time compared to YJ0
SYJ-331	CEN.PK 113-11C	PYJ-331	*TcGPAT3*, *TcLPAT3* and *TcDGAT1* gene combination expression
SYJ-332	CEN.PK 113-11C	PYJ-332	*TcGPAT3*, *TcLPAT3* and *TcDGAT2* gene combination expression
SYJ-341	CEN.PK 113-11C	PYJ-341	*TcGPAT3*, *TcLPAT4* and *TcDGAT1* gene combination expression
SYJ-342	CEN.PK 113-11C	PYJ-342	*TcGPAT3*, *TcLPAT4* and *TcDGAT2* gene combination expression
SYJ-431	CEN.PK 113-11C	PYJ-431	*TcGPAT4*, *TcLPAT3* and *TcDGAT1* gene combination expression
SYJ-432	CEN.PK 113-11C	PYJ-432	*TcGPAT4*, *TcLPAT3* and *TcDGAT2* gene combination expression
SYJ-441	CEN.PK 113-11C	PYJ-441	*TcGPAT4*, *TcLPAT4* and *TcDGAT1* gene combination expression
SYJ-442	CEN.PK 113-11C	PYJ-442	*TcGPAT4*, *TcLPAT4* and *TcDGAT2* gene combination expression
Y29	IMX581 [19]	None	IMX581 *sct1*Δ*ale1*Δ *lro1*Δ *dga1*Δ
Y29-pBS01A	Y29	pBS01A	Empty plasmid pBS01A
Y29-TcD1	Y29	PYJ-TcDGAT1	*TcDGAT1 expression*
Y29-TcD2	Y29	PYJ-TcDGAT2	*TcDGAT2 expression*
Y29-331	Y29	PYJ-331	*TcGPAT3*, *TcLPAT3* and *TcDGAT1* gene combination expression
Y29-441	Y29	PYJ-441	*TcGPAT4*, *TcLPAT4* and *TcDGAT1* gene combination expression
YJW01	CEN.PK 113-11C	None	CEN.PK 113-11C *sct1*Δ
YJW02	CEN.PK 113-11C	None	CEN.PK 113-11C *sct1*Δ::*LsGPAT*
YJW09	CEN.PK 113-11C	None	CEN.PK 113-11C *sct1*Δ::*LsGPAT gpt2*Δ

Yeast strains were selected on synthetic complete (SC) dropout medium (Formedium Ltd) or YPD medium (10 g l^−1^. Bacto yeast extract, 20 g l^−1^ Bacto peptone and 20 g l^−1^ glucose) (Merck Millipore or Difco) containing 200 mg ml^−1^ G418. *L*. *starkeyi* was cultivated on YPD medium. Minimal medium (7.5 g l^−1^ (NH_4_)_2_SO_4_, 14.4 g l^−1^ KH_2_PO_4_, 0.5 g l^−1^ MgSO_4_·7H_2_O, 20 g l^−1^ glucose, trace metal solution and vitamin solution), supplemented with 100 mg l^−1^ histidine or 200 mg l^−1^ G418 (Formedium), was used for 20 ml shake flask batch cultivations [20, 21]. A nitrogen-limited medium (named NLM medium in the text) [22] was used for large-scale (1 l) shake flask batch cultivations.

### Cocoa sample collection

The cocoa fruit samples were collected from the same tree in the Gothenburg Botanical Garden in a nearly ripe (May 26, 2015) and unripe (October 12, 2015) state, respectively (Additional file 1: Figure S2). One ripe cocoa fruit and several unripe cocoa fruits including small and big fruits were collected, respectively. After collection, the cocoa samples were cut into small pieces and immediately put into liquid nitrogen. The samples were kept at − 80 °C before use.

### RNA preparation and cDNA synthesis

Cocoa fruits were grinded to fine powder in liquid N_2_ using mortar and pestle, and the fine powder was divided into 100 mg aliquots in 1.5 ml Eppendorf tubes. Subsequently, 0.5 ml cold (4 °C) PureLink plant RNA reagent (Life Technologies) was added to each tube, mixed briefly by vortexing until the sample was thoroughly resuspended, and then the tubes were incubated for 5 min at room temperature. The resulting solution was cleared by centrifuging at 12,000*g* in a microcentrifuge for 2 min at room temperature. Next, the lysate was transferred to a QIAshredder spin column placed in a 2 ml collection tube and the instructions of the RNeasy plant mini kit (Qiagen) were followed to obtain total RNA. For extraction of RNA from *L*. *starkeyi*, the yeast was cultivated in YPD medium for 48 h at 30 °C and 200 rpm, and the biomass was collected by centrifugation at 12,000 rpm for 1 min. The yeast RNA was extracted using the Qiagen RNeasy mini kit. The cocoa and yeast RNA was transcribed into cDNA by reverse transcriptase using the Qiagen QuantiTect Reverse Transcription Kit.

### Phylogenetic analysis of cocoa GPAT, LPAT and DGAT genes

The GPAT and LPAT gene sequences of *T*. *cacao* were downloaded from the Genbank database. Reference GPAT and LPAT genes sequences of *Arabidopsis thaliana*, *Homo sapiens* and *S. cerevisiae* were downloaded from the KEGG database [23]. Amino acid sequences of GPATs, LPATs or DGATs were aligned using the MAFFT online version [24], and the multiple alignment results were used to create phylogenetic trees using the MEGA 7.0.21 software [25]. The Neighbor-Joining method with Poisson correction was used to create the tree and the bootstrap confidence values were based on 1000 replicates. Moreover, gaps in the alignment of GPAT and LPAT sequences were treated with the pairwise deletion option.

### Plasmid and yeast strain construction

Cocoa genes encoding GPATs and LPATs were amplified using primers described in Additional file 1: Table S1 from cocoa fruit cDNA using the PrimeSTAR HS DNA polymerase (Takara) according to the manufacturer’s instruction. The one GPAT and one LPAT sequences amplified from cocoa cDNA, which were different from the available annotated genes, were deposited at the GenBank database under the accession numbers MF352000-MF352001. The primers used to amplify cocoa genes, promoters and terminators are listed in Additional file 1: Table S1 and some primers used for cocoa gene combination expression were the same as described before [9]. The *LsGPAT* gene was cloned from *L*. *starkeyi* cDNA using PrimeSTAR HS DNA polymerase (Takara) and the sequence was the same as described before [26].

**Fig. 2:**
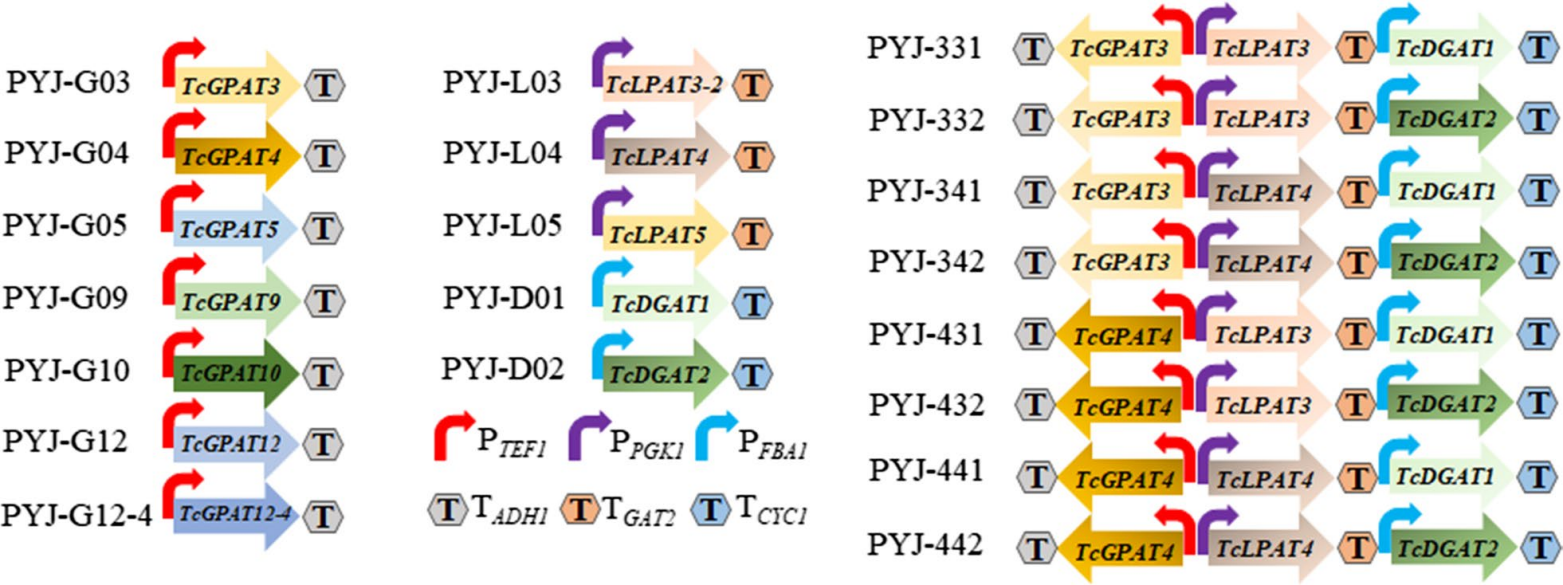
Schematic organization of cocoa gene expression cassettes in each of the expression plasmids

The cocoa gene expression cassettes were verified by sequencing and illustrated in Fig. 2. Gibson assembly (NEB) was used to construct cocoa gene expression plasmids by ligation of the gene expression cassettes and the amplified linear backbone fragment of plasmid pBS01A, and were further verified by PCR and Sanger sequencing (Additional file 1: Table S2). The constructed plasmids were used to transform *S. cerevisiae* to construct the strains listed in Table 1.

### Shake flask cultivation and lipid analysis

Shake flask fermentations were carried out in 20 ml minimal medium and cultivated at 30 °C and 200 rpm for 24 h. [9, 27]. Each strain was cultivated in three replicates, and the fatty acid methyl ester (FAME) and lipid profiles were analyzed using a microwave-assisted method [28, 29]. In order to obtain sufficient lipids for TAG analyses, 5 l shake flasks containing 1 l NLM medium were used for yeast biomass collection [9, 27]. The lipids extracted from each strain were used to analyze yeast TAG profiles by UPLC using RI detection [30]. The TAG compositions of each strain were expressed in relative area percentages [27, 30]. CB standards and TAG composition sequences were completed by AAK, and TAG standards were purchased from Larodan by AAK.

## Results

### Seven cocoa GPAT and three cocoa LPAT were cloned from cocoa cDNA

**Fig. 3:**
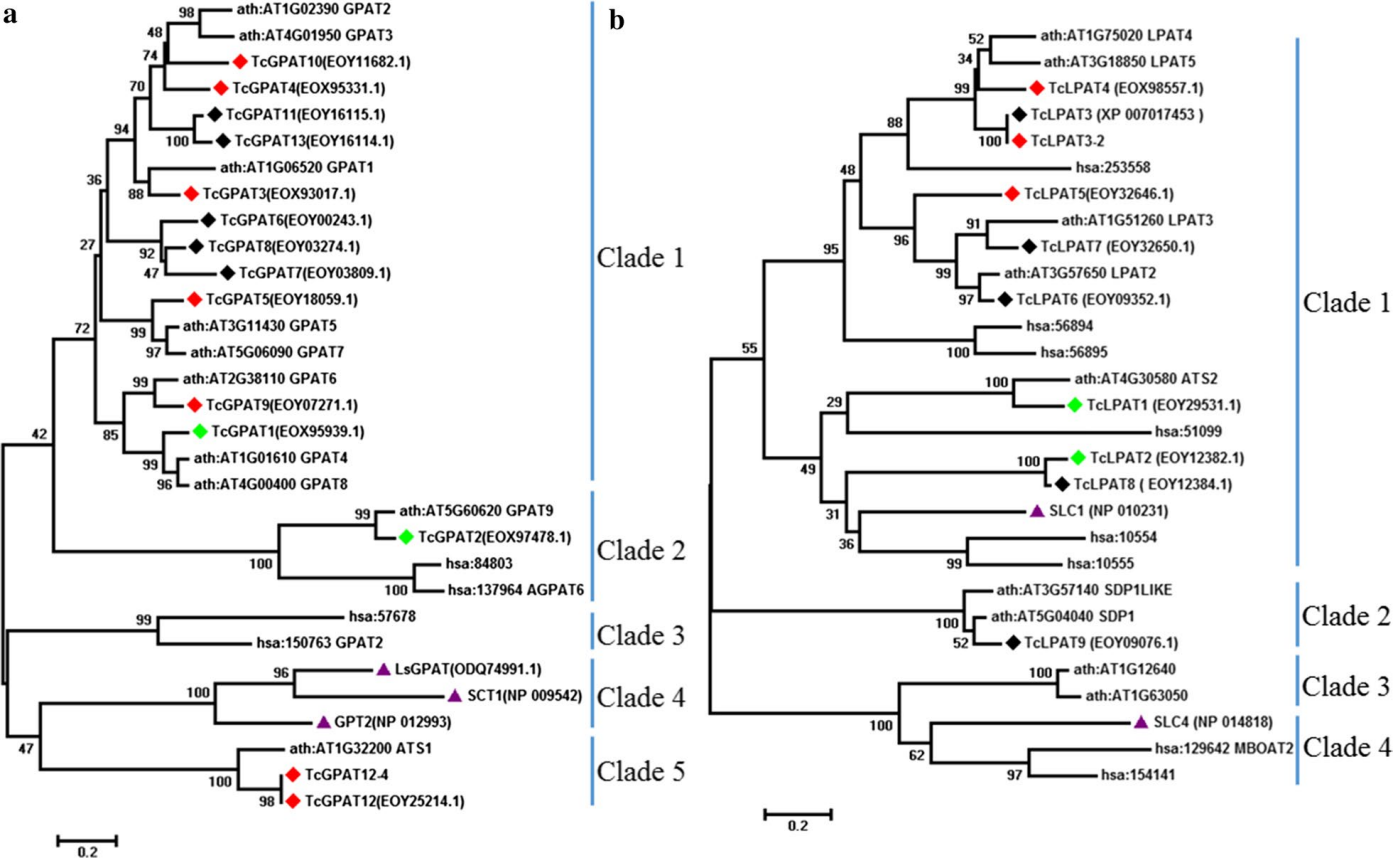
Phylogenetic analysis of GPAT (**a**) and LPAT (**b**) amino acid sequences with an unrooted tree. All neighbor-joining trees were constructed using the MEGA 7.0.21 software (bootstrap values: 1000) with the peptide sequences. Cocoa genes are marked with rhombuses, genes marked with red rhombuses are genes cloned from cDNA of *T. cacao* in this study, genes marked with green rhombuses are genes synthesized in a previous study, genes marked with black rhombuses are genes which were not cloned or synthesized in this study; yeast genes are marked with purple triangles; genes of *A. thaliana* and *H. sapiens* are not marked. The bootstrap values are marked above the nodes and the scale bar is indicated under each tree

Usually, there are many GPAT and LPAT genes in one plant species, and 13 genes were annotated as GPAT genes and nine genes were annotated as LPAT genes (*TcLPAT10* was removed as a result of standard genome annotation processing) in *T. cacao* [16, 17]. To characterize potential GPAT and LPAT genes, we designed primers to clone all the possible GPAT and LPAT genes except two GPAT (*TcGPAT1* and *TcGPAT2*) and two LPAT (*TcLPAT1* and *TcLPAT2*) genes, which had been synthesized and characterized in yeast before [9]. A total of seven GPAT and three LPAT genes were cloned from cocoa fruit cDNA. Most of the gene sequences were consistent with the genes described in the genomic data, except two genes, *TcGPAT12*-*4* and *TcLPAT3*-*2*, which were different from the published corresponding gene sequences (Fig. 3). *TcGPAT12*-*4* and *TcLPAT3*-*2* were 231 and 114 bp shorter than *TcGPAT12* and *TcLPAT3*, respectively, this might be due to the alternative splicing in plants [31]. Though we tried several different PCR conditions to clone more cocoa GPAT and LPAT genes, no other genes were further cloned from cocoa cDNA samples.

All cocoa GPAT sequences and their reference sequences can be divided into five clades in a phylogenetic tree, and the cocoa GPAT sequences were distributed in four clades except Clade 4 which contained yeast GPAT genes (Fig. 3a). The highest level of identity between the amino acids sequence of any cocoa GPAT gene and the yeast GPAT genes is less than 15%. At the same time, the identities between yeast GPAT genes and the *Lipomyces starkeyi* GPAT gene are 45% (Gpt2p and LsGPAT) and 41% (Sct1p and LsGPAT), respectively (Fig. 3a). We successfully cloned *TcGPAT3*, *TcGPAT4*, *TcGPAT5*, *TcGPAT9*, *TcGPAT10*, and *TcGPAT12*. *TcGPAT2* had been characterized before, so that each clade had at least one representative cloned or synthesized cocoa GPAT sequence (Fig. 3a). The LPAT sequences can be divided into four clades, and the cocoa genes *TcLPAT3*, *TcLPAT4* and *TcLPAT5* all formed part of LPAT clade 1 (Fig. 3b). The two cocoa DGAT genes, *TcDGAT1* and *TcDGAT2*, have been characterized before. Additional annotated DGATs (*TcDGAT3*-*TcDGAT11*) were similar with wax ester synthase genes of *A*. *thaliana*, hinting they would not participate in TAG biosynthesis [9].

### Expression of single cocoa genes in *S. cerevisiae* changed its total fatty acid production

**Fig. 4:**
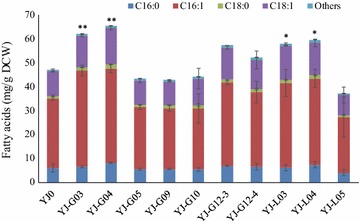
Total fatty acid production in different *S. cerevisiae* strains expressing single cocoa genes. Others represents the summed content of C12:0, C14:0, C14:1, C20:0, C20:1, C22:0, C24:0 and C26:0 fatty acids. The error bars represent the standard deviation of three biological replicates. Asterisks (*) indicate significant difference between the yeast strains harboring cocoa genes and control strain YJ0. *p < 0.05; **p < 0.01. The p values are calculated based on paired t tests corrected for multiple comparisons

The cloned cocoa genes were assembled in expression cassettes with strong constitutive promoters and ligated into plasmid pBS01A as described in Fig. 2 and Additional file 1: Table S2. The empty plasmid pBS01A and 10 other plasmids harboring cocoa genes were introduced into *S. cerevisiae* CEN.PK 113-11C, resulting in the control strain YJ0 and another 10 yeast strains, respectively (Table 1). Fatty acid analysis showed that the most abundant fatty acids in each strain were C16 and C18 fatty acids and some yeast strains harboring cocoa genes produced more fatty acids than the control strain YJ0 (Fig. 4). Especially, yeast strains YJ-G03, YJ-G04, YJ-L03 and YJ-L04 displayed a significant increase of the total fatty acid amount compared to YJ0, indicating that *TcGPAT3*, *TcGPAT4*, *TcLPAT3*-*2* and *TcLPAT4* were active in the yeast and might be engaged in cocoa CB biosynthesis.

### Expression of several cocoa gene combinations in *S. cerevisiae* altered lipid production and compositions

**Fig. 5:**
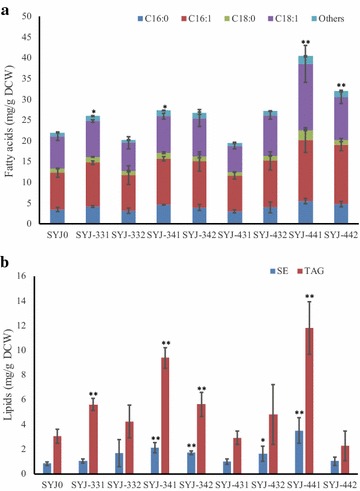
Total fatty acid (**a**) and lipid (**b**) production of *S. cerevisiae* strains harboring combinations of cocoa genes. *SE* steryl esters. Asterisks (*) of indicate significant differences of fatty acids (**a**) and lipids (**b**) between strains harboring the empty plasmid and strains harboring cocoa genes; *p < 0.05; **p < 0.01. The p values are calculated based on paired t tests corrected for multiple comparisons

Six cocoa genes (including the four cDNA-derived cocoa genes *TcGPAT3*, *TcGPAT4*, *TcLPAT3*-*2*, *TcLPAT4*, and the two cocoa DGAT genes *TcDGAT1* and *TcDGAT2*) were assembled in eight different combinations to generate 8 additional yeast strains (Table 1). Among all these strains, four (SYJ-331, SYJ-341, SYJ-441 and SYJ-442) displayed a significant increase of total fatty acids (19–84% increase) over the control strain SYJ0 (Fig. 5a). For C16 and C18 fatty acids of the eight strains, C16:0, C16:1 and C18:0 contents of SYJ-331, C18:0 and C18:1 contents of SYJ-432, C16:0, C16:1, C18:0 and C18:1 contents of SYJ-441, and C16:0, C16:1 and C18:1 contents of SYJ-442 increased and showed significant difference compared with SYJ0.

The neutral lipids steryl esters (SE) and TAG are the two main storage lipids in *S. cerevisiae*, whose production is affected by the expression of GPAT, LPAT and DGAT genes [14, 32, 33]. The SE content analysis showed that SYJ-341, SYJ-342 and SYJ-441 displayed a significant increase over the control strain SYJ0, while the TAG content analysis showed that SYJ-331, SYJ-341, SYJ-342 and SYJ-441 displayed a significant increase over the control (Fig. 5b), suggesting expression of some cocoa gene combinations were beneficial for yeast storage lipid production. Among the eight strains, SYJ-341 and SYJ-441 produced 150 and 320% more SE than SYJ0, respectively, and they also produced 210% and 290% more TAG than SYJ0, respectively.

### Cocoa gene expression partially restored neutral lipid production in a *S. cerevisiae* mutant with a partial TAG biosynthesis pathway

**Fig. 6:**
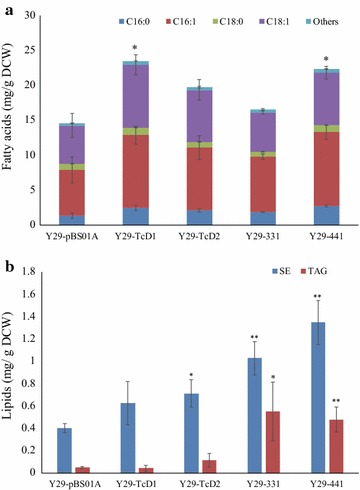
Total fatty acid (**a**) and lipid (**b**) production of Y29 strains harboring an empty plasmid (Y29-pBS01A) or plasmids with cocoa genes. *SE* steryl esters. Asterisks (*) indicate significant differences in comparison to the control strain; *p < 0.05; **p < 0.01. The p values are calculated based on paired t tests corrected for multiple comparisons

To further verify if the cloned cocoa GPAT and LPAT genes functioned in *S. cerevisiae*, we deleted parts of the yeast TAG biosynthetic pathway, including the GPAT gene *SCT1*, the LPAT gene *ALE1*, the DGAT gene *DGA1* and the PDAT gene *LRO1*, to construct new yeast strain Y29 and expressed TAG biosynthetic genes in it (Table 1). Compared with the control strain SYJ0, the SE content was reduced, but specifically the TAG content drastically decreased (Figs. 5b, 6b). With regards to total fatty acids, Y29-TcD1 and Y29-441 displayed a significant difference with the control Y29-pBS01A, and the concentration of C16:0 in Y29-TcD2 and Y29-331 increased and showed significant differences with the control (Fig. 6a). Concerning the neutral lipids, SE and TAG in Y29-331 and Y29-441 displayed significant increase over the control strain. Besides, SE in Y29-TcD2 also showed significant increase compared to the control strain (Fig. 6b). Moreover, we also attempted to delete one yeast GPAT gene (*SCT1* or *GPT2*) and replace the other gene with one cocoa GPAT gene, but we failed to obtain viable colonies. However, when we used *LsGPAT* of *L. starkeyi* to replace yeast *SCT1* in the yeast strain carrying the *GPT2* deletion, it succeeded and yeast fatty acid and lipid production changed, indicating that a single of the cloned or previously synthesized cocoa GPAT gene cannot replace the function of yeast GPAT genes (Additional file 1: Figure S3). Unfortunately, the CBL production of yeast strain YJW09 was lower than the wild-type strain, though the CBL content of *L starkeyi* is higher than that of *S. cerevisiae* (Additional file 1: Figure S3B) [27].

### Expression of cocoa gene combinations led to an increase in potential CBL production

We compared TAG production of four different yeast strains: control strain SYJ0, SYJ-331, which displayed a significantly higher fatty acid and TAG content compared to SYJ0, SYJ-342, which had an increased SE and TAG content, and SYJ-441, which displayed the highest amounts of total fatty acids, SE and TAG of the eight strains. The TAG compositions changed after introducing the cocoa genes in *S. cerevisiae* (Additional file 1: Figure S4A). Though all the four yeast strains produced more than 20 different kinds of TAGs, most of the TAGs only accounted for less than 5% of the total TAGs (Additional file 1: Figure S4A).

**Fig. 7:**
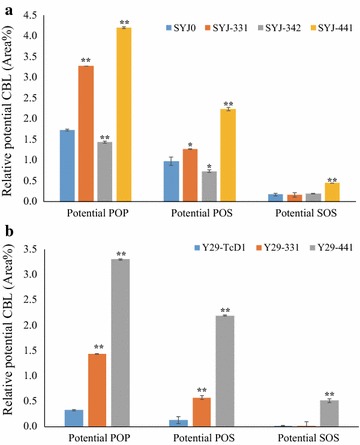
Relative potential CBL content of *S. cerevisiae* CEN.PK 113-11C-derived strains (**a**) and Y29-derived strains (**b**) harboring cocoa genes. Shown is the peak area of the respective TAG in comparison to the summed peak areas of all TAGs. The error bars represent the standard deviation of two biological replicates. Asterisks (*) indicate significant differences in comparison to control strain SYJ0 (**a**) or Y29-TcD1 (**b**); *p < 0.05; **p < 0.01. The p values are calculated based on paired t tests corrected for multiple comparisons

Concerning the relative CBL production, SYJ-331 (4.7%) and SYJ-441 (6.9%) produced 64 and 140% more potential CBL than the control strain SYJ0 (2.87%), respectively, but SYJ-342 produced less CBL than the control (Fig. 7a). The potential POP and POS of SYJ-331 and SYJ-441 had increased by at least 30% and by at least 130%, respectively. The potential SOS (C18:0, C18:1, C18:0) proportion of SYJ-441 displayed a significant difference compared with SYJ0 (Fig. 7a). In fact, the potential POP, POS and SOS proportions of SYJ-441 had increased 143, 130 and 164%, respectively, compared with SYJ0. Considering that SYJ-441 produced 288% more TAGs than SYJ0, its potential CBL production increased more than eightfold compared with SYJ0, showing this cocoa gene combination of *TcGPAT4*, *TcLPAT4* and *TcDGAT1* not only increased TAG production, but also helped *S. cerevisiae* accumulate more potential CBL.

As the TAG content of Y29-pBS01A was quite low, only TAG compositions in Y29-TcD1, Y29-331 and Y29-441 were further checked and all of them could produce more than 17 different kinds of TAGs (Additional file 1: Figure S4B). The potential CBL proportion had increased from 0.48% of Y29-TcD1 to 2.02% in Y29-331 and to 6.01% in Y29-441, which means an increase of 321 and 1150%, respectively (Fig. 7b). For details, the potential POP and POS in Y29-331 had increased 334 and 331% compared with Y29-TcD1, respectively; the potential POP, POS and SOS in Y29-441 had increased 898, 1543 and 3012% compared with Y29-TcD1 (Fig. 7b). Considering the TAG content in Y29-331 (0.55 mg g^−1^) and Y29-441 (0.48 mg g^−1^) was 11.5-fold and 9.8-fold higher than in Y29-TcD1 (Fig. 6b), the potential CBL content in Y29-331 and Y29-441 was 51-fold and 134-fold higher than in Y29-TcD1. The significant increase of potential CBL proportion in Y29-331 and Y29-441 over in Y29-TcD1 further suggested that cocoa GPAT and LPAT genes functioned in yeast and helped accumulate more TAGs.

### Expression of cocoa genes in different yeast strains changed fatty acid profiles and TAG composition

**Fig. 8:**
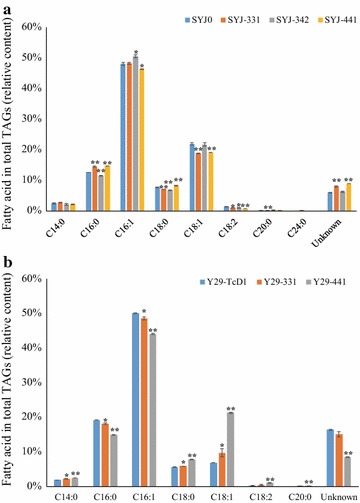
Fatty acid composition of the TAGs of *S. cerevisiae* CEN.PK 113-11C-derived (**a**) and Y29-derived (**b**) strains. The error bars represent the standard deviation of two biological replicates. Shown is the peak area of the respective TAG in comparison to the summed peak areas of all TAGs. Asterisks (*) indicate significant differences in comparison to reference strain SYJ0 (**a**) or Y29-TcD1 (**b**), respectively; *p < 0.05; **p < 0.01. The p values are calculated based on paired t tests corrected for multiple comparisons

The C16 and C18 fatty acids were the main fatty acids in the TAGs of four yeast strains (SYJ0, SYJ-331, -342 and -441) and their proportions were similar, which is consistent with the total fatty acid composition analyses (Figs. 5a, 6a). Generally, saturated fatty acids in the TAGs of SYJ-331 and -441 increased, but decreased in the TAGs of SYJ-342 (Fig. 8a). Compared with SYJ0, for SYJ-331, the C16:0 portion in the TAGs increased, while other C16 and C18 fatty acids in the TAGs were equal or decreased; for SYJ-342, the C16:1 fraction in the TAGs increased, while other C16 and C18 fatty acids in the TAGs were equal or decreased; for SYJ-441, both C16:0 and C18:0 fatty acids in the TAGs increased with a significant difference, while other C16 and C18 fatty acids in the TAGs were equal or decreased.

Though C16 and C18 fatty acids were the main fatty acids in the TAGs of three Y29 strains, the C16 fatty acid faction decreased in Y29-331 and Y29-441 and C18 fatty acids showed an increase in the TAGs of Y29-331 and Y29-441 compared with Y29-TcD1 (Fig. 8b). Besides, saturated fatty acids in the TAGs of Y29-331 and Y29-441 decreased compared with Y29-TcD1. In details, C14:0, C16:0, C16:1 fractions in the TAGs of Y29-331 and -441 decreased, while C18:0 and C18:1 increased. Besides, the C18:2 and C20:0 content in the TAGs of Y29-441 increased. However, the content of unknown TAGs was higher in these three Y29 strains (Y29-TcD1, Y29-331 and Y29-441) than in the four yeast strains (SYJ0, SYJ-331, -342 and -441) and the TAG profiles of Y29-derived strains were different from the other four yeast strains, further suggesting that cocoa GPAT and LPAT enzymes were active and altered the TAG profiles in the yeast strains (Fig. 8).

## Discussion

CB is mainly extracted from cocoa beans, but the genes of its biosynthetic pathway in cocoa tree are unknown, hampering their application for developing microbial based CBL production [1, 16, 17]. 13 GPAT, 9 LPAT and 11 DGAT genes have been identified in the *T*. *cacao* genome [9, 16, 17]. Of the DGAT genes, only *TcDGAT1* and *TcDGAT2* are similar to the yeast DGAT genes, the others are similar to *LRO1* of *S. cerevisiae* or wax ester synthase genes, and they might not participate in CB production. Two GPAT (*TcGPAT1* and *TcGPAT2*), two LPAT (*TcLPAT1* and *TcLPAT2*) and two DGAT (*TcDGAT1* and *TcDGAT2*) genes of *T*. *cacao* have been synthesized and characterized before [9]. In this study, we cloned another seven GPAT genes and three LPAT genes from seeds of *T*. *cacao*. Expression of some of these genes (*TcGPAT3*, *TcGPAT4*, *TcLPAT3*-*2* and *TcLPAT4*) in yeast resulted in an increased total fatty acid production, indicating that these cocoa genes might play roles in cocoa CB production. Furthermore, expression of cocoa gene combinations including these genes plus the previously characterized DGAT genes (*TcGPAT3*, *TcGPAT4*, *TcLPAT3*-*2*, *TcLPAT4*, *TcDGAT1* and *TcDGAT2*) increased fatty acid, neutral lipid and CBL production significantly, suggesting that cocoa GPATs and LPATs functioned in yeast and contributed to a CBL production increase in this heterologous host.

The esterification of acyl-CoAs to the glycerol backbone during TAG biosynthesis is catalyzed by GPAT, LPAT and DGAT [13]. Our results showed that SE and TAG production of strain Y29 decreased drastically, demonstrating that deletion of yeast TAG biosynthesis genes altered total neutral lipid production [11, 15]. Compared with Y29-derived strains harboring either an empty plasmid or only one of the cocoa DGAT genes (Y29-TcD1 and Y29-TcD2), expression of cocoa gene combinations (Y29-331 and Y29-441) restored part of yeast neutral lipid production and CBL production, suggesting cocoa GPAT and LPAT contributed to the yeast CBL production increase. Furthermore, both GPAT genes of *S. cerevisiae* were replaced by *LsGPAT* of *L. starkeyi*, showing that the GPAT genes of *S. cerevisiae* can be replaced by homologues from the same clade (Clade 4 in Fig. 3a). However, replacement of both yeast GPAT genes with one cocoa GPAT gene (at least under the conditions used in this study) failed, suggesting that overexpression of one cocoa GPAT gene cannot complement the loss of both GPAT genes in *S. cerevisiae*. Moreover, neutral lipid production of strain Y29 harboring one cocoa GPAT gene, one cocoa LPAT gene and one cocoa DGAT gene was much lower than the wild-type yeast strains, further demonstrating cocoa TAG biosynthetic gene expression cannot compensate the gene loss in *S. cerevisiae*. The low identities between cocoa GPAT genes and yeast GPAT genes might be the reason why cocoa GPAT genes cannot replace yeast GPAT genes (Fig. 3a). As the model plant *Arabidopsis thaliana* contains several genes encoding enzymes with GPAT activities, the situation for the cocoa tree might be similar, pointing to that several genes/enzymes may function simultaneously in vivo [34].

YJ0 and SYJ0 are supposed to have the same genotype, but when we cultivated these two strains in the same conditions (but not in the same experiment), the fatty acid production of YJ0 and SYJ0 was different. This might be due to the fact that YJ0 and SYJ0 were constructed at different time points and/or that they were in a different growth state when sampled. Overexpression of cocoa TAG biosynthetic genes increased the CBL content from 2.87% in the wild-type yeast strain SYJ0 to 6.9% in SYJ-441. In addition, overexpression of cocoa TAG biosynthetic genes in Y29 increased the CBL content, and the highest CBL content of Y29-441 was 6.0%. With regard to the less than 5.4% CBL content in the yeast strain YJ-221 harboring specifically selected cocoa TAG biosynthetic genes [9], this demonstrates that combination of phylogenetic analyses and experimental verification can lead to identification of effective enzymes (*TcGPAT4* and *TcLPAT4*) for CBL production. The relative CBL content in SYJ-441 is still low (only 6.9%) and the relative CBL content in YJW09 harboring *LsGPAT* is even lower than in the wild-type *S. cerevisiae* strain, hinting that other metabolic engineering strategies should be attempted to increase yeast CBL production, including an improvement of specific fatty acid supply and the identification of other functional TAG biosynthetic genes from plants [35–38]. Considering that the C16:1 proportion in the yeast TAGs is more than 40% and that the C16:1 content in CB is very low [1], some strategies should be further implemented to alter the total fatty acid profiles of the yeast, such as screening for heterologous desaturases which can decrease C16:1 production and increase C18:1 production. Besides, the C16:1 content in the TAGs of some of the Y29-derived strains harboring cocoa gene combinations was lower than in the control strain of Y29-TcD1 (Fig. 8b), which is another indication that these cocoa genes are potential CB biosynthetic genes. As several GPAT, LPAT or DGAT genes might be responsible for CB production in vivo simultaneously, overexpression of different cocoa TAG biosynthetic genes in one yeast strain would be a potential strategy to increase CBL production further. In the future, also overexpression of the identified genes in oleaginous yeasts represents a possibility to obtain a high-level of CBL production [27].

## Conclusions

Ten different cocoa TAG biosynthetic genes were cloned from cDNA of *T. cacao* and characterized in yeast. Expression of some cocoa genes in a wild-type *S. cerevisiae* strain increased CBL production more than eightfold (the relative CBL content is 6.9%) over the wild-type strain, and expression in yeast mutant strain with a reduced TAG pathway could increase CBL production 134-fold (the relative CBL content is 6.0%) over the control strain Y29-TcD1. Besides, our results demonstrate that several cocoa GPAT, LPAT and DGAT genes might function simultaneously in *T. cacao* for CB production.

## Additional file

**Additional file 1: Figure S1.** Gene cassettes used for gene deletion (A) and gene replacement (B) in CEN.PK 113-11C. Up means upstream sequence of the targeted gene; Down means downstream sequence of the targeted gene; Gene fragment means part of the targeted gene. **Figure S2.** The cocoa fruit samples in nearly ripe (May 26, 2015) (A) and unripe (October 12, 2015) (B) state at Gothenburg Botanical Garden. **Figure S3.** Total fatty acid production (A) and relative TAG content (B) in four different *S. cerevisiae* strains. (A) Others represents the summed content of C12:0, C14:0, C14:1, C20:0, C20:1, C22:0, C24:0 and C26:0 fatty acids. The error bars represent the standard deviation of biological replicates. (B) All TAGs identified in the four yeast strains are shown. The error bars represent the standard deviation of two biological replicates. Shown is the peak area of the respective TAG in comparison to the summed peak areas of all TAGs. Asterisks (*) indicate a significant difference between the yeast strains and the control CEN.PK 113-11C. * indicates p < 0.05; ** indicates p < 0.01. The p-values are calculated based on paired t-tests corrected for multiple comparisons. **Figure S4.** Relative TAG content (except potential CBL) of CEN.PK 113-11C-derived strains (A) and Y29-derived strains (B). (A) Relative TAG content (except potential CBL) of CEN.PK 113-11C-derived strains harboring cocoa genes. The error bars represent the standard deviation of two biological replicates. Shown is the peak area of the respective TAG in comparison to the summed peak areas of all TAGs. Asterisks (*) indicate significant differences (p-values are based on paired t-tests corrected for multiple comparisons) in comparison to control SYJ0. * indicates p < 0.05; ** indicates p < 0.01. (B) Relative TAG content (except potential CBL) of Y29-derived strains harboring 3 cocoa genes or 1 cocoa gene. Asterisks (*) indicate significant differences (p-values are based on paired t-tests corrected for multiple comparisons) in comparison to reference strain Y29-TcD1. * indicates p < 0.05; ** indicates p < 0.01. **Table S1.** List of primers used in this study. **Table S2.** List of plasmids constructed in this study.

## Abbreviations

CB: cocoa butter
CBL: CB-like lipids
TAG: triacylglycerol
POP: 1,3-dipalmitoyl-2-oleoyl-glycerol (C16:0-C18:1-C16:0)
POS: 1-palmitoyl-3-stearoyl-2-oleoyl-glycerol (C16:0-C18:1-C18:0)
SOS: 1,3-distearoyl-2-oleoyl-glycerol (C18:0-C18:1-C18:0)
GPAT: glycerol-3-phosphate acyltransferase
LPAT: lysophospholipid acyltransferase
DGAT: diacylglycerol acyltransferase
Acyl-CoA: acyl-coenzyme A
DAG: diacylglycerol
SE: steryl esters
