# Supplementary material for: Expression of cocoa genes in Saccharomyces cerevisiae improves cocoa butter production
Source: Microb Cell Fact. 2018 Jan 25;17:11. doi: 10.1186/s12934-018-0866-2 (PMC5784687; doi:10.1186/s12934-018-0866-2)
Supplement: Supplementary file 1 — Additional file 1: Figure S1. Gene cassettes used for gene deletion (A) and gene replacement (B) in CEN.PK 113-11C. Up means upstream sequence of the targeted gene; Down means downstream sequence of the targeted gene; Gene fragment means part of the targeted gene. Figure S2. The cocoa fruit samples in nearly ripe (May 26, 2015) (A) and unripe (October 12, 2015) (B) state at Gothenburg Botanical Garden. Figure S3. Total fatty acid production (A) and relative TAG content (B) in four different S. cerevisiae strains. (A) Others represents the summed content of C12:0, C14:0, C14:1, C20:0, C20:1, C22:0, C24:0 and C26:0 fatty acids. The error bars represent the standard deviation of biological replicates. (B) All TAGs identified in the four yeast strains are shown. The error bars represent the standard deviation of two biological replicates. Shown is the peak area of the respective TAG in comparison to the summed peak areas of all TAGs. Asterisks (*) indicate a significant difference between the yeast strains and the control CEN.PK 113-11C. * indicates p < 0.05; ** indicates p < 0.01. The p-values are calculated based on paired t-tests corrected for multiple comparisons. Figure S4. Relative TAG content (except potential CBL) of CEN.PK 113-11C-derived strains (A) and Y29-derived strains (B). (A) Relative TAG content (except potential CBL) of CEN.PK 113-11C-derived strains harboring cocoa genes. The error bars represent the standard deviation of two biological replicates. Shown is the peak area of the respective TAG in comparison to the summed peak areas of all TAGs. Asterisks (*) indicate significant differences (p-values are based on paired t-tests corrected for multiple comparisons) in comparison to control SYJ0. * indicates p < 0.05; ** indicates p < 0.01. (B) Relative TAG content (except potential CBL) of Y29-derived strains harboring 3 cocoa genes or 1 cocoa gene. Asterisks (*) indicate significant differences (p-values are based on paired t-tests corrected for multipl [file 12934_2018_866_MOESM1_ESM.docx]

**Expression of cocoa genes in *Saccharomyces cerevisiae* improves cocoa butter production**

**Yongjun Wei^1,2,4^,** **David Bergenholm^1,2^, Michael Gossing^1,2^, Verena Siewers^1,2^ and Jens Nielsen^1,2,3^****^*^**

**^1^** Department of Biology and Biological Engineering, Chalmers University of Technology, SE-41296 Gothenburg, Sweden

**^2^** Novo Nordisk Foundation Center for Biosustainability, Chalmers University of Technology, SE-41296 Gothenburg, Sweden

**^3^** Novo Nordisk Foundation Center for Biosustainability, Technical University of Denmark, DK-2800 Kgs. Lyngby, Denmark

^4^ Present address: CAS-Key Laboratory of Synthetic Biology, Institute of Plant Physiology and Ecology, Shanghai Institutes for Biological Sciences, Chinese Academy of Sciences, Shanghai 200032, China.

**E-mail:**

Yongjun Wei: [weyongjun@163.com](mailto:weyongjun@163.com)

David Bergenholm: david.bergenholm@chalmers.se

Michael Gossing: gossing@chalmers.se

Verena Siewers: siewers@chalmers.se

Jens Nielsen: [nielsenj@chalmers.se](mailto:nielsenj@chalmers.se)

**^*^Corresponding author**

Jens Nielsen

E-mail: [nielsenj@chalmers.se](mailto:nielsenj@chalmers.se)

Telephone: +46 (0)31 772 3804

Fax: +46(0)31 772 3801

**
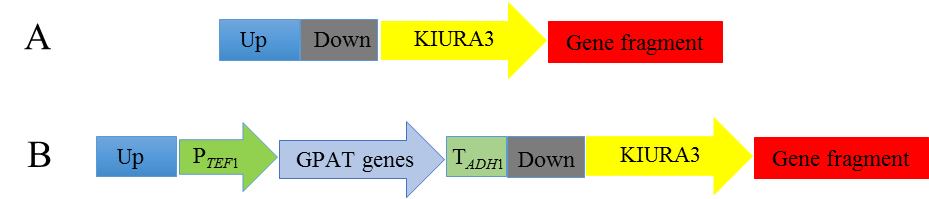
**

Figure S1, Gene cassettes used for gene deletion (A) and gene replacement (B) in CEN.PK 113-11C. Up means upstream sequence of the targeted gene; Down means downstream sequence of the targeted gene; Gene fragment means part of the targeted gene.

**
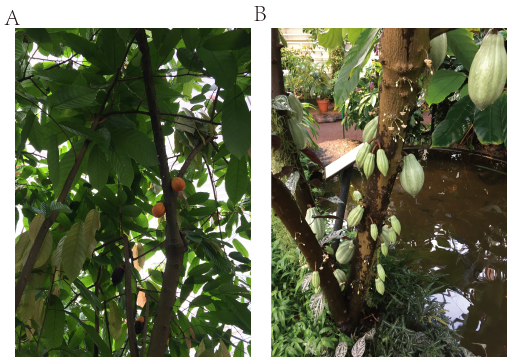
**

Figure S2, The cocoa fruit samples in nearly ripe (May 26, 2015) (A) and unripe (October 12, 2015) (B) state at Gothenburg Botanical Garden.

**
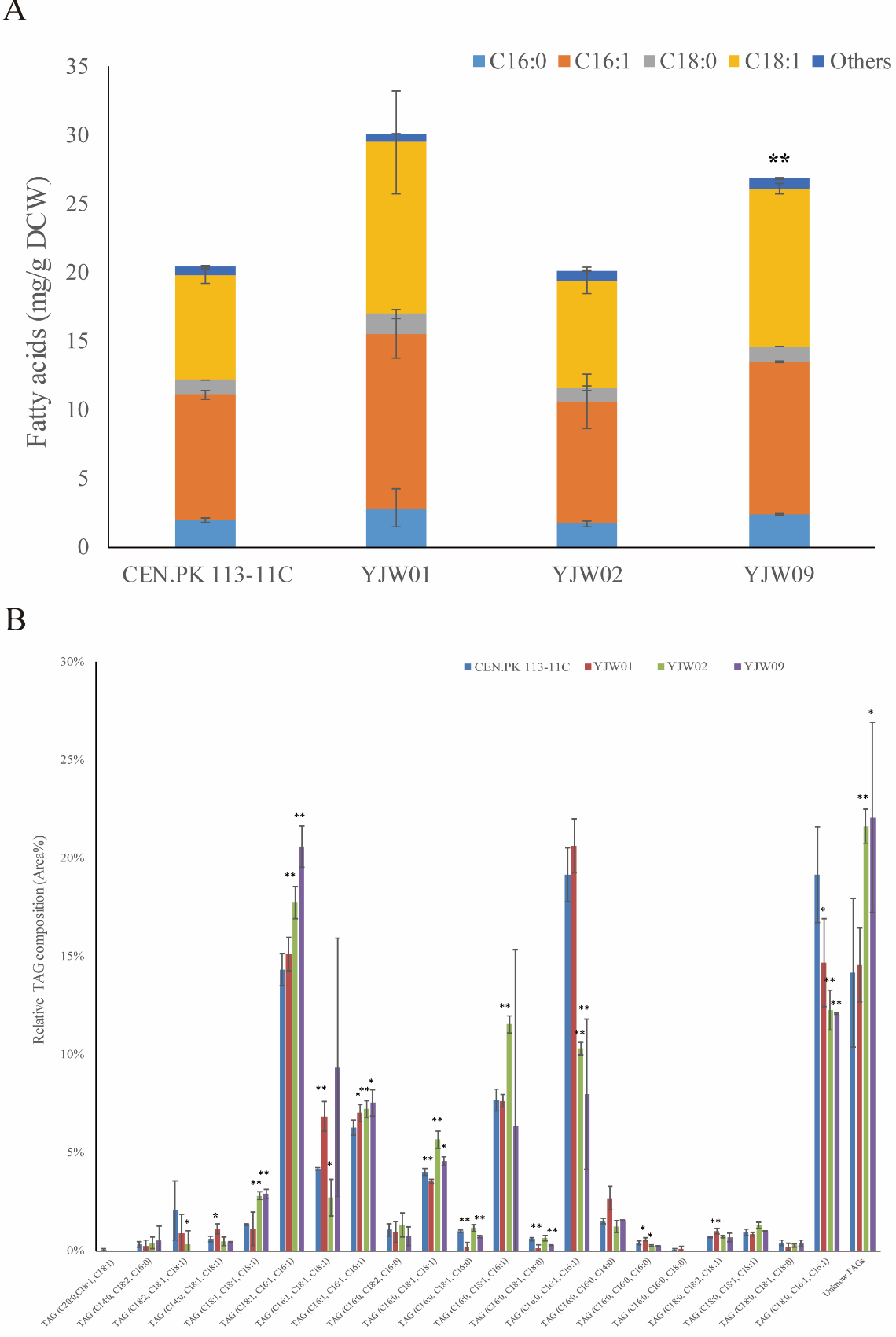
**

Figure S3, Total fatty acid production (A) and relative TAG content (B) in four different *S. cerevisiae* strains. (A) Others represents the summed content of C12:0, C14:0, C14:1, C20:0, C20:1, C22:0, C24:0 and C26:0 fatty acids. The error bars represent the standard deviation of biological replicates. (B) All TAGs identified in the four yeast strains are shown. The error bars represent the standard deviation of two biological replicates. Shown is the peak area of the respective TAG in comparison to the summed peak areas of all TAGs. Asterisks (*) indicate a significant difference between the yeast strains and the control CEN.PK 113-11C. * indicates p<0.05; ** indicates p<0.01. The p-values are calculated based on paired t-tests corrected for multiple comparisons.


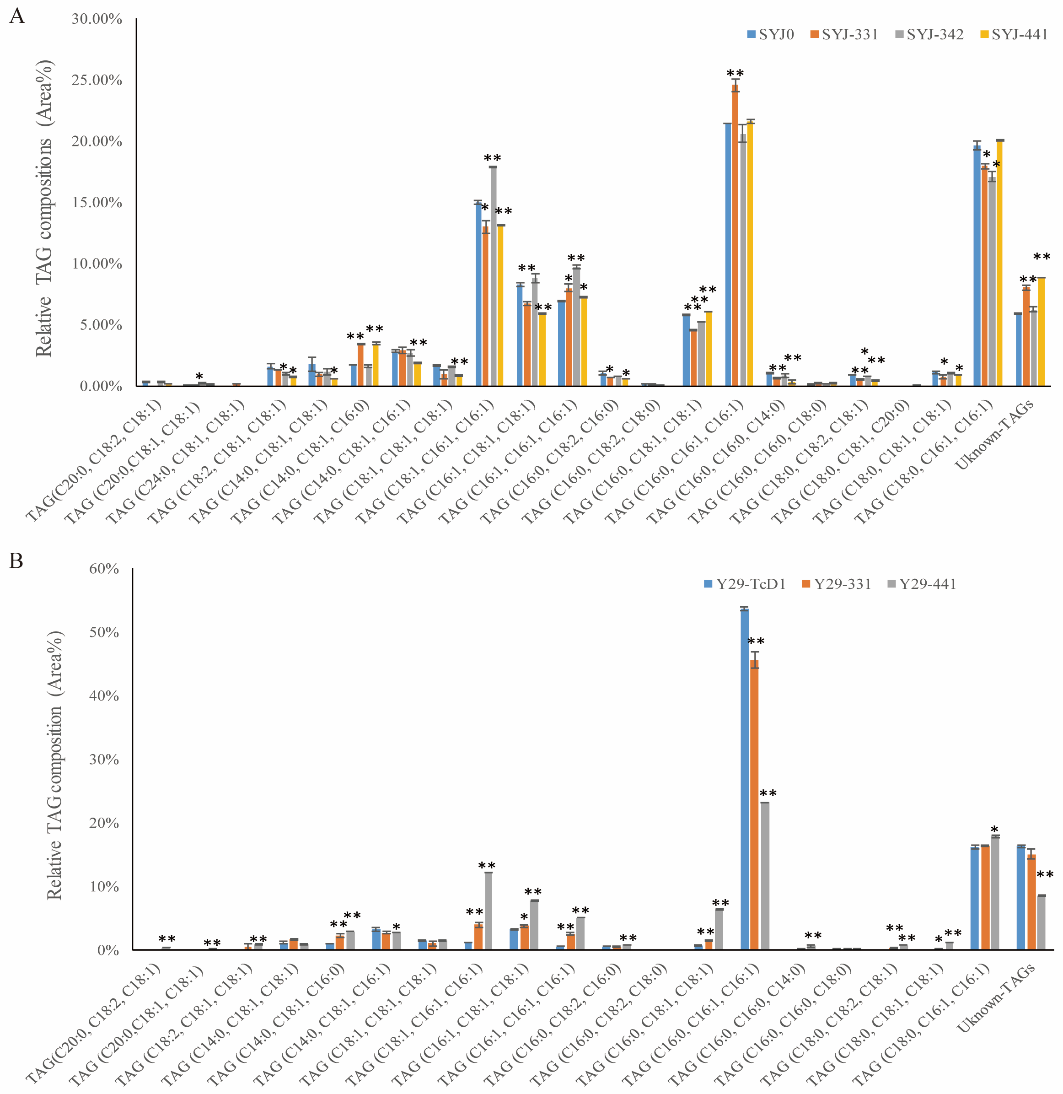


Figure S4, Relative TAG content (except potential CBL) of CEN.PK 113-11C-derived strains (A) and Y29-derived strains (B). (A) Relative TAG content (except potential CBL) of CEN.PK 113-11C-derived strains harboring cocoa genes. The error bars represent the standard deviation of two biological replicates. Shown is the peak area of the respective TAG in comparison to the summed peak areas of all TAGs. Asterisks (*) indicate significant differences (p-values are based on paired t-tests corrected for multiple comparisons) in comparison to control SYJ0. * indicates p<0.05; ** indicates p<0.01. (B) Relative TAG content (except potential CBL) of Y29-derived strains harboring 3 cocoa genes or 1 cocoa gene. Asterisks (*) indicate significant differences (p-values are based on paired t-tests corrected for multiple comparisons) in comparison to reference strain Y29-TcD1. * indicates p<0.05; ** indicates p<0.01.

**Table S1 List of primers used in this study.**

| **Name** | **Sequence(5'-3')** | **Application** |
| --- | --- | --- |
| **DGA1_targetRNA fw** | **TGCGCATGTTTCGGCGTTCGAAACTTCTCCGCAGTGAAAGATAAATGATCAAATGATTAACAACATCATCGTTTTAGAGCTAGAAATAGCAAGTTAAAATAAGGCTAGTCCGTTATCAAC** | **gRNA cassette construction** |
| **DGA1_targetRNA rv** | **GTTGATAACGGACTAGCCTTATTTTAACTTGCTATTTCTAGCTCTAAAACGATGATGTTGTTAATCATTTGATCATTTATCTTTCACTGCGGAGAAGTTTCGAACGCCGAAACATGCGCA** | **gRNA cassette construction** |
| **DGA1_repair oligo fw** | **CACATACACTTACATATACATAAGGAAACGCAGAGGCATACAGTTTGAACAGTCACATAATAATGAATTCATTGGAAAACACAAAATATGTTAGAATAAATAAGGATTTTTTAGTGTTTG** | **Repair oligos** |
| **DGA1_repair oligo rv** | **CAAACACTAAAAAATCCTTATTTATTCTAACATATTTTGTGTTTTCCAATGAATTCATTATTATGTGACTGTTCAAACTGTATGCCTCTGCGTTTCCTTATGTATATGTAAGTGTATGTG** | **Repair oligos** |
| **DGA1_dg fw** | **GAAGTACTTCACCACGGGGG** | **Diagnostic primers for seamless deletion confirmation** |
| **DGA1_dg rv** | **GCCTCTCAGTTACGCTTTGC** | **Diagnostic primers for seamless deletion confirmation** |
| **LRO1_targetRNA fw** | **TGCGCATGTTTCGGCGTTCGAAACTTCTCCGCAGTGAAAGATAAATGATCGATTCTGATGAAAACAATAAGTTTTAGAGCTAGAAATAGCAAGTTAAAATAAGGCTAGTCCGTTATCAAC** | **gRNA cassette construction** |
| **LRO1_targetRNA rv** | **GTTGATAACGGACTAGCCTTATTTTAACTTGCTATTTCTAGCTCTAAAACTTATTGTTTTCATCAGAATCGATCATTTATCTTTCACTGCGGAGAAGTTTCGAACGCCGAAACATGCGCA** | **gRNA cassette construction** |
| **LRO1_repair oligo fw** | **ATAGTAACAGCCATTACAAAAGGTTCTCTACCAACGAATTCGGCGACAATCGAGTAAAAAATGACCGACATTGACTCACTATCCATCCGTGTATTATTTCAAAGAGCGAAAAGAAGGCGC** | **Repair oligos** |
| **LRO1_repair oligo rv** | **GCGCCTTCTTTTCGCTCTTTGAAATAATACACGGATGGATAGTGAGTCAATGTCGGTCATTTTTTACTCGATTGTCGCCGAATTCGTTGGTAGAGAACCTTTTGTAATGGCTGTTACTAT** | **Repair oligos** |
| **LRO1_dg fw** | **TCTGGCCCTTCAACTTCGAC** | **Diagnostic primers for seamless deletion confirmation** |
| **LRO1_dg rv** | **GTACGTCGCTGCTGCAATTG** | **Diagnostic primers for seamless deletion confirmation** |
| **SCT1_targetRNA fw** | **TGCGCATGTTTCGGCGTTCGAAACTTCTCCGCAGTGAAAGATAAATGATCCAAGTCTACAGTTAAAATAAGTTTTAGAGCTAGAAATAGCAAGTTAAAATAAGGCTAGTCCGTTATCAAC** | **gRNA cassette construction** |
| **SCT1_targetRNA rv** | **GTTGATAACGGACTAGCCTTATTTTAACTTGCTATTTCTAGCTCTAAAACTTATTTTAACTGTAGACTTGGATCATTTATCTTTCACTGCGGAGAAGTTTCGAACGCCGAAACATGCGCA** | **gRNA cassette construction** |
| **SCT1_repair oligo fw** | **GCCCGGAATTAAATATATAGTAAAAAGAGCACAGGGGCGTTTACATCGGGGTAAAAAAAAATGCCATTTACTGACGGTGAAGATACTAGAAACTAAATCTTTCGCCGTTCTATTTATGTA** | **Repair oligos** |
| **SCT1_repair oligo rv** | **TACATAAATAGAACGGCGAAAGATTTAGTTTCTAGTATCTTCACCGTCAGTAAATGGCATTTTTTTTTACCCCGATGTAAACGCCCCTGTGCTCTTTTTACTATATATTTAATTCCGGGC** | **Repair oligos** |
| **SCT1_dg fw** | **AAGTAGGCCCGCCTTCTTTC** | **Diagnostic primers for seamless deletion confirmation** |
| **SCT1_dg rv** | **CGGCTACTGGTCTCTTCCTG** | **Diagnostic primers for seamless deletion confirmation** |
| **ALE1_targetRNA fw** | **TGCGCATGTTTCGGCGTTCGAAACTTCTCCGCAGTGAAAGATAAATGATCTATTTACGTGTCACAAAGAAGTTTTAGAGCTAGAAATAGCAAGTTAAAATAAGGCTAGTCCGTTATCAAC** | **gRNA cassette construction** |
| **ALE1_targetRNA rv** | **GTTGATAACGGACTAGCCTTATTTTAACTTGCTATTTCTAGCTCTAAAACTTCTTTGTGACACGTAAATAGATCATTTATCTTTCACTGCGGAGAAGTTTCGAACGCCGAAACATGCGCA** | **gRNA cassette construction** |
| **ALE1_repair oligo fw** | **CAAACCGCATACGCCAAGACAAACCGTGGTGATTTAATTCTGCTGCTGATCGCTTCCAACATGGCCAAAAACGACAGATGCGTGTGGAAGTCACAGTCTTGTTGTCTTATTTTCCAGTTC** | **Repair oligos** |
| **ALE1_repair oligo rv** | **GAACTGGAAAATAAGACAACAAGACTGTGACTTCCACACGCATCTGTCGTTTTTGGCCATGTTGGAAGCGATCAGCAGCAGAATTAAATCACCACGGTTTGTCTTGGCGTATGCGGTTTG** | **Repair oligos** |
| **ALE1_dg fw** | **GTCGAAACCACAAACCGTCG** | **Diagnostic primers for seamless deletion confirmation** |
| **ALE1_dg rv** | **ACCGTACCGCCATCTCAAAG** | **Diagnostic primers for seamless deletion confirmation** |
| **GPT2_targetRNA fw** | **TGCGCATGTTTCGGCGTTCGAAACTTCTCCGCAGTGAAAGATAAATGATCAATTATTCACTTAAAAAAACGTTTTAGAGCTAGAAATAGCAAGTTAAAATAAGGCTAGTCCGTTATCAAC** | **gRNA cassette construction** |
| **GPT2_targetRNA rv** | **GTTGATAACGGACTAGCCTTATTTTAACTTGCTATTTCTAGCTCTAAAACGTTTTTTTAAGTGAATAATTGATCATTTATCTTTCACTGCGGAGAAGTTTCGAACGCCGAAACATGCGCA** | **gRNA cassette construction** |
| **GPT2_repair oligo fw** | **AATTCTACTTTCACATCTAATAAAGCCGATTAATCGATCAGTTATTGCTCCCTTTCCTTTTCAAAAAATAGAAAAATAAAAAAAAGCATTTGACAATGTTTGTAAATAATATTATGAAAG** | **Repair oligos** |
| **GPT2_repair oligo rv** | **CTTTCATAATATTATTTACAAACATTGTCAAATGCTTTTTTTTATTTTTCTATTTTTTGAAAAGGAAAGGGAGCAATAACTGATCGATTAATCGGCTTTATTAGATGTGAAAGTAGAATT** | **Repair oligos** |
| **GPT2_dg fw** | **TTCGTGCTTCGTCGCTAGAG** | **Diagnostic primers for seamless deletion confirmation** |
| **GPT2_dg rv** | **TCTTGGCTAGGACGGCATTG** | **Diagnostic primers for seamless deletion confirmation** |
| **SLC1_targetRNA fw** | **TGCGCATGTTTCGGCGTTCGAAACTTCTCCGCAGTGAAAGATAAATGATCTAACATGAAGATATCCAAGGGTTTTAGAGCTAGAAATAGCAAGTTAAAATAAGGCTAGTCCGTTATCAAC** | **gRNA cassette construction** |
| **SLC1_targetRNA rv** | **GTTGATAACGGACTAGCCTTATTTTAACTTGCTATTTCTAGCTCTAAAACCCTTGGATATCTTCATGTTAGATCATTTATCTTTCACTGCGGAGAAGTTTCGAACGCCGAAACATGCGCA** | **gRNA cassette construction** |
| **SLC1_repair oligo fw** | **AATTCTTCAATAGAGAAGTTTAGTGGTTTCCCTCCGTCAGTGAATTCGAGCAAAAAAATAGCCACCACCACATTTTTAGAGTAGTATATAGACCCAAAAACTGTAATTATCTTTTTAAAA** | **Repair oligos** |
| **SLC1_repair oligo rv** | **TTTTAAAAAGATAATTACAGTTTTTGGGTCTATATACTACTCTAAAAATGTGGTGGTGGCTATTTTTTTGCTCGAATTCACTGACGGAGGGAAACCACTAAACTTCTCTATTGAAGAATT** | **Repair oligos** |
| **SLC1_dg fw** | **AGCGATGAGATGCGACTCTG** | **Diagnostic primers for seamless deletion confirmation** |
| **SLC1_dg rv** | **GTCGAGGAGGTTCTGCCATC** | **Diagnostic primers for seamless deletion confirmation** |
| **TcGPAT3-fw** | **ACATCCGAACATAAACAACCATGGTTTTCCCTGTGGTATTTCTG** | **Cocoa gene cloning** |
| **TcGPAT3-rv** | **ATAAATCATAAGAAATTCGCTTAATTTCTCTTATTTTCATGGACAACC** | **Cocoa gene cloning** |
| **TcGPAT4-fw** | **ACATCCGAACATAAACAACCATGGCTAAACTTTCTATGGAGTTTTC** | **Cocoa gene cloning** |
| **TcGPAT4-rv** | **ATAAATCATAAGAAATTCGCTTAGGTTTGGCTAATTATTCCTTCGT** | **Cocoa gene cloning** |
| **TcGPAT5-fw** | **ACATCCGAACATAAACAACCATGGAATCAGTTGTTTCTGAGCTG** | **Cocoa gene cloning** |
| **TcGPAT5-rv** | **ATAAATCATAAGAAATTCGCTCATTGAAAAAAAGGCTTAAAAGTGCTC** | **Cocoa gene cloning** |
| **TcGPAT6-fw** | **ACATCCGAACATAAACAACCATGAAACTTAGCCGACGCCAGAC** | **Cocoa gene cloning** |
| **TcGPAT6-rv** | **ATAAATCATAAGAAATTCGCTTAACAGCCCATTACTTTGTTGGCTG** | **Cocoa gene cloning** |
| **TcGPAT7-fw** | **ACATCCGAACATAAACAACCATGGGATCCAGGATCTTGCCG** | **Cocoa gene cloning** |
| **TcGPAT7-rv** | **ATAAATCATAAGAAATTCGCCTACGTCTTTGTTCCTTCAGGG** | **Cocoa gene cloning** |
| **TcGPAT8-fw** | **ACATCCGAACATAAACAACCATGGCCAAAACAAAAGAAAACCC** | **Cocoa gene cloning** |
| **TcGPAT8-rv** | **ATAAATCATAAGAAATTCGCCTAGCACCCCATGACCTTATT** | **Cocoa gene cloning** |
| **TcGPAT9-fw** | **ACATCCGAACATAAACAACCATGGTCATGGGGGCTCACC** | **Cocoa gene cloning** |
| **TcGPAT9-rv** | **ATAAATCATAAGAAATTCGCTTAAGCTTTTTCCTTTTCCTGTTC** | **Cocoa gene cloning** |
| **TcGPAT10-fw** | **ACATCCGAACATAAACAACCATGGCTGGAAAACCACAAAACC** | **Cocoa gene cloning** |
| **TcGPAT10-rv** | **ATAAATCATAAGAAATTCGCCTAGACTATTCCCTGGTTCCC** | **Cocoa gene cloning** |
| **TcGPAT11-fw** | **ACATCCGAACATAAACAACCATGGCCAACCGACAATCAGTG** | **Cocoa gene cloning** |
| **TcGPAT11-rv** | **ATAAATCATAAGAAATTCGCCTATACGATCCCCTCATTGCCT** | **Cocoa gene cloning** |
| **TcGPAT12-fw** | **ACATCCGAACATAAACAACCATGTCTACTTTGCCTCTCCCATTC** | **Cocoa gene cloning** |
| **TcGPAT12-rv** | **ATAAATCATAAGAAATTCGCCTAGTTCCATGGCTGTGACAAAG** | **Cocoa gene cloning** |
| **TcLPAT3-fw** | **TTTACAACAAATATAAAACA****ATGGAAGTTTGCAGGCCCCTC** | **Cocoa gene cloning** |
| **TcLPAT3-rv** | **TATGAATGTATGATTTTATATTATTCATCTCTCTTGCCCTTTGG** | **Cocoa gene cloning** |
| **TcLPAT4-fw** | **TTTACAACAAATATAAAACAATGGAAGTTCCTAGTGCAAATCATG** | **Cocoa gene cloning** |
| **TcLPAT4-rv** | **TATGAATGTATGATTTTATATTAGACACAAGTTTTCACAAGGCTG** | **Cocoa gene cloning** |
| **TcLPAT5-fw** | **TTTACAACAAATATAAAACAATGGAAATTTCTGCCATAGTTACTAG** | **Cocoa gene cloning** |
| **TcLPAT5-rv** | **TATGAATGTATGATTTTATATTATGTATCTCCATTTGTCTGCAAAG** | **Cocoa gene cloning** |
| **TcLPAT6-fw** | **TTTACAACAAATATAAAACAATGGCGATTGCAGCGGCAG** | **Cocoa gene cloning** |
| **TcLPAT6-rv** | **TATGAATGTATGATTTTATACTACTGCTGTTTGTCCCGTC** | **Cocoa gene cloning** |
| **TcLPAT7-fw** | **TTTACAACAAATATAAAACAATGGCAATCCCAGCTGCAC** | **Cocoa gene cloning** |
| **TcLPAT7-rv** | **TATGAATGTATGATTTTATATTAACAGAGAAGCTTTTCTTTTATTG** | **Cocoa gene cloning** |
| **TcLPAT8-fw** | **TTTACAACAAATATAAAACAATGGAGAGTTCTGGAAGTGG** | **Cocoa gene cloning** |
| **TcLPAT8-rv** | **TATGAATGTATGATTTTATATTATGATCTTGAACTGTTAGTGGTG** | **Cocoa gene cloning** |
| **TcLPAT9-fw** | **TTTACAACAAATATAAAACAATGGAAATAAGTAATGAGGCCAGG** | **Cocoa gene cloning** |
| **TcLPAT9-rv** | **TATGAATGTATGATTTTATATCAACCATCCACAACACCCC** | **Cocoa gene cloning** |
| **TcGPAT6-fw** | **ATGAAACTTAGCCGACGCCAGACAGT** | **Cocoa gene cloning** |
| **TcGPAT6-rv** | **TTAACAGCCCATTACTTTGTTGGCTGAAAAC** | **Cocoa gene cloning** |
| **TcGPAT7-fw** | **ATGGGATCCAGGATCTTGCCGTC** | **Cocoa gene cloning** |
| **TcGPAT7-rv** | **CTACGTCTTTGTTCCTTCAGGGCAT** | **Cocoa gene cloning** |
| **TcGPAT11-fw** | **ATGGCCAACCGACAATCAGTGCTG** | **Cocoa gene cloning** |
| **TcGPAT11-rv** | **CTATACGATCCCCTCATTGCCTGC** | **Cocoa gene cloning** |
| **TcLPAT6-fw** | **ATGGCGATTGCAGCGGCAGCT** | **Cocoa gene cloning** |
| **TcLPAT6-rv** | **CTACTGCTGTTTGTCCCGTCTTG** | **Cocoa gene cloning** |
| **TcLPAT7-fw** | **ATGGCAATCCCAGCTGCACTTG** | **Cocoa gene cloning** |
| **TcLPAT7-rv** | **TTAACAGAGAAGCTTTTCTTTTATTGAATC** | **Cocoa gene cloning** |
| **TcLPAT8-fw** | **ATGGAGAGTTCTGGAAGTGGTTC** | **Cocoa gene cloning** |
| **TcLPAT8-rv** | **TTATGATCTTGAACTGTTAGTGGTGTC** | **Cocoa gene cloning** |
| **TcLPAT9-fw** | **ATGGAAATAAGTAATGAGGCCAGGG** | **Cocoa gene cloning** |
| **TcLPAT9-rv** | **TCAACCATCCACAACACCCCGATC** | **Cocoa gene cloning** |
| **TcLPAT3S-fw** | **TTTACAACAAATATAAAACAATGTTTCTAGCGTATTTAGGACCTG** | **Cocoa gene cloning** |
| **pBS01A-pTEF-fw** | **CAGTTATTACCCGCGATCGCGACATGGAGGCCCAGAATAC** | **Cocoa gene expression cassette construction** |
| **pBS01A-pTEF-R** | **GGTTGTTTATGTTCGGATGTGATG** | **Cocoa gene expression cassette construction** |
| **pBS01A-tADH-fw** | **GCGAATTTCTTATGATTTATGATTTTTAT** | **Cocoa gene expression cassette construction** |
| **pBS01A-tADH-R** | **CTAACAACAACAACCTCGAGCCGGTAGAGGTGTGGTCA** | **Cocoa gene expression cassette construction** |
| **pBS01A-pPGK-fw** | **CAGTTATTACCCGCGATCGCCTGGAAGTACCTTCAAAGAATG** | **Cocoa gene expression cassette construction** |
| **pBS01A-pPGK-R** | **TGTTTTATATTTGTTGTAAAAAGTAGATAAT** | **Cocoa gene expression cassette construction** |
| **pBS01A-tGAT2-fw** | **TATAAAATCATACATTCATATAATATCCAT** | **Cocoa gene expression cassette construction** |
| **pBS01A-tGAT2-R** | **CTAACAACAACAACCTCGAGGGGAAAACGTTAGGAAAACG** | **Cocoa gene expression cassette construction** |
| **pBS01A-pFBA1-fw** | **CAGTTATTACCCGCGATCGCCACTGGTAGAGAGCGACTTTG** | **Cocoa gene expression cassette construction** |
| **pBS01A-pFBA1-R** | **TTTGAATATGTATTACTTGGTTATGGTT** | **Cocoa gene expression cassette construction** |
| **pBS01A-tCYC1-fw** | **ACAGGCCCCTTTTCCTTTGT** | **Cocoa gene expression cassette construction** |
| **pBS01A-tCYC1-R** | **CTAACAACAACAACCTCGAGTTTGTACAGAAAAAAAAGAAAAATTTGAAAT** | **Cocoa gene expression cassette construction** |
| **pBS01-fw** | **CTCGAGGTTGTTGTTGTTAGATCT** | **Cocoa gene expression cassette construction** |
| **pBS01-rv** | **GCGATCGCGGGTAATAACTGAT** | **Cocoa gene expression cassette construction** |
| **Ls-GPAT-fw** | **ACATCCGAACATAAACAACCATGGCTTCCTCAGTTCCTCAAG** | **Cloning of GPAT gene of *L. starkeyi*** |
| **Ls-GPAT-rv** | **ATAAATCATAAGAAATTCGCCTAACTCTGCATTAACCGGTGAGAG** | **Cloning of GPAT gene of *L. starkeyi*** |
| **SceSCT1-up-fw** | **TTCATGAGTGTAAAGTGATGAGAGACGC** | ***SCT1* replacement and deletion primer** |
| **SceSCT1-up-rv** | **CTGGGCCTCCATGTCTTTTTTTTACCCCGATGTAAACGCCCCT** | ***SCT1* replacement primer** |
| **SceSCT1_pTEF1-GPAT-fw** | **TCGGGGTAAAAAAAAGACATGGAGGCCCAGAATACCCT** | ***SCT1* replacement primer** |
| **SceSCT1-tADH1-GPAT-rv** | **TCACCGTCAGTAAATGGCATCCGGTAGAGGTGTGGTCAATAAGAG** | ***SCT1* replacement primer** |
| **SceSCT1-down-fw** | **CCTCTACCGGATGCCATTTACTGACGGTGAAGAT** | ***SCT1* replacement primer** |
| **SceSCT1-down-rv** | **CCAGAAACTACATCATCGAAGGATGAAGAATCTTTACGCCAATAC** | ***SCT1* replacement and deletion primer** |
| **KIURA-fw** | **TTCGATGATGTAGTTTCTGGT** | ***SCT1* replacement primer** |
| **KIURA-rv** | **GTGATTCTGGGTAGAAGATCG** | ***SCT1* replacement primer** |
| **SceSCT1-fragment-fw** | **GATCTTCTACCCAGAATCACGAGACCACTGGTTTTATCTCTTACAT** | ***SCT1* replacement and deletion primer** |
| **SceSCT1-fragment-rv** | **CTACGCATCTCCTTCTTTCCCTTC** | ***SCT1* replacement and deletion primer** |
| **SceSCT1-deletion-up-rv** | **GTCAGTAAATGGCATTTTTTTTTACCCCGATGTAAACGCCCCT** | ***SCT1* deletion primer** |
| **SceSCT1-deletion-down-fw** | **TCGGGGTAAAAAAAAATGCCATTTACTGACGGTGAAGAT** | ***SCT1* deletion primer** |
| **SceGPT2-up-fw** | **GCGAGATCTAGCTGCGGGAGAAATC** | ***GPT2* replacement and deletion primer** |
| **SceGPT2-up-rv** | **CTGGGCCTCCATGTCAAAGGAAAGGGAGCAATAACTGATCG** | ***GPT2* replacement primer** |
| **SceGPT2_pTEF1-GPAT-fw** | **TGCTCCCTTTCCTTTGACATGGAGGCCCAGAATACCCT** | ***GPT2* replacement primer** |
| **SceGPT2-tADH1-GPAT-rv** | **TTTATTTTTCTATTTTTTGACCGGTAGAGGTGTGGTCAATAAGAG** | ***GPT2* replacement primer** |
| **SceGPT2-down-fw** | **CCTCTACCGGTCAAAAAATAGAAAAATAAAAAAAAGCATTTG** | ***GPT2* replacement primer** |
| **SceGPT2-down-rv** | **CCAGAAACTACATCATCGAAAGTGATCGTAGCTGTGATTACC** | ***GPT2* replacement and deletion primer** |
| **SceGPT2-fragment-fw** | **GATCTTCTACCCAGAATCACGCTGTACGATGTGTCTGTATTTCTG** | ***GPT2* replacement and deletion primer** |
| **SceGPT2-fragment-rv** | **TCTGAGTATACGCGACAAATAATG** | ***GPT2* replacement and deletion primer** |
| **SceGPT2-deletion-up-rv** | **TTTTCTATTTTTTGAAAAGGAAAGGGAGCAATAACTGATCG** | ***GPT2* deletion primer** |
| **SceGPT2-deletion-down-fw** | **CCTTTCCTTTTCAAAAAATAGAAAAATAAAAAAAAGCATTTGAC** | ***GPT2* deletion primer** |

**Table S2 List of plasmids constructed in this study.**

| **Name** | **Parent plasmid** | **Properties** | **Reference** |
| --- | --- | --- | --- |
| **pBS01A** | **pSP-GM1** | **Expression empty plasmid, *amp* (ampicillin resistance), *URA3*** | **Provided by Anastasia Krivoruchko** |
| **PYJ-G03** | **pBS01A** | ***TcGPAT3* expression** | **This study** |
| **PYJ-G04** | **pBS01A** | ***TcGPAT4 expression*** | **This study** |
| **PYJ-G05** | **pBS01A** | ***TcGPAT5 expression*** | **This study** |
| **PYJ-G09** | **pBS01A** | ***TcGPAT9 expression*** | **This study** |
| **PYJ-G10** | **pBS01A** | ***TcGPAT10 expression*** | **This study** |
| **PYJ-G12** | **pBS01A** | ***TcGPAT12 expression*** | **This study** |
| **PYJ-G12-4** | **pBS01A** | ***TcGPAT12-4 expression*** | **This study** |
| **PYJ-L03** | **pBS01A** | ***TcLPAT3 expression*** | **This study** |
| **PYJ-L04** | **pBS01A** | ***TcLPAT4 expression*** | **This study** |
| **PYJ-L05** | **pBS01A** | ***TcLPAT5 expression*** | **This study** |
| **PYJ-331** | **pBS01A** | ***TcGPAT3*, *TcLPAT3* and *TcDGAT1* gene combination expression** | **This study** |
| **PYJ-332** | **pBS01A** | ***TcGPAT3*, *TcLPAT3* and *TcDGAT2* gene combination expression** | **This study** |
| **PYJ-341** | **pBS01A** | ***TcGPAT3*, *TcLPAT4* and *TcDGAT1* gene combination expression** | **This study** |
| **PYJ-342** | **pBS01A** | ***TcGPAT3*, *TcLPAT4* and *TcDGAT2* gene combination expression** | **This study** |
| **PYJ-431** | **pBS01A** | ***TcGPAT4*, *TcLPAT3* and *TcDGAT1* gene combination expression** | **This study** |
| **PYJ-432** | **pBS01A** | ***TcGPAT4*, *TcLPAT3* and *TcDGAT2* gene combination expression** | **This study** |
| **PYJ-441** | **pBS01A** | ***TcGPAT4*, *TcLPAT4* and *TcDGAT1* gene combination expression** | **This study** |
| **PYJ-442** | **pBS01A** | **TcGPAT4, *TcLPAT4* and *TcDGAT2* gene combination expression** | **This study** |
